# Supplementary material for: Alleviation of Aflatoxin B1-Induced Hepatic Damage by Propolis: Effects on Inflammation, Apoptosis, and Cytochrome P450 Enzyme Expression
Source: Curr Issues Mol Biol. 2026 Jan 1;48(1):56. doi: 10.3390/cimb48010056 (PMC12840301; doi:10.3390/cimb48010056)
Supplement: Supplementary file 1 [file cimb-48-00056-s001.zip › cimb-4053332-supplementary.pdf]

Supplementary Table S1

LC/MS/MS analysis of phenolic compounds in propolis.

Quantitative Analysis Summary Report					
**Batch Data Path**	D:\MassHunter\Data\2023\06-Haziran\Phenolic_Compounds\QuantResults\Phenolic_Compounds_Propolis.batch.				
**Analysis Time**	6/16/2023 5:23 PM	**Analyst Name**	LCMS\admin		
**Report Time**	6/16/2023 5:24 PM	**Reporter Name**	LCMS\admin		
**Last Calib Update**	6/16/2023 5:23 PM	**Batch State**	Processed		
**Quant Batch Version**	B.07.01	**Quant Report Version**	B.07.01		
**Sequence Table**					
**Data File**	**Acq Method File**	**Sample Name**	**Sample Type**	**Position**	**Volume**
SK_Propolis-Num.d	Phenolic_Compounds.m	SK_Propolis-Num	Sample	P1-B5	-1.00
**Quantitation Results**					
**Data File**	**Compound**	**Sample Type**	**Response**	**Final Conc**	**Unit**
SK_Propolis-Num.d	Quinic Acid	Sample	7080	2683.9697	ng/mL
SK_Propolis-Num.d	Fumaric Acid	Sample	4028	2389.1864	ng/mL
SK_Propolis-Num.d	Gallic Acid	Sample	4919	147.9120	ng/mL
SK_Propolis-Num.d	Pyrogallol	Sample	5	0.0000	ng/mL
SK_Propolis-Num.d	Keracyanin Chloride	Sample	6171	1845.1999	ng/mL
SK_Propolis-Num.d	Cyanidin-3-o-glucoside	Sample	18030	1819.2552	ng/mL
SK_Propolis-Num.d	Chlorogenic Acid	Sample	45995	1718.2447	ng/mL
SK_Propolis-Num.d	Catechin	Sample	542	17.1751	ng/mL
SK_Propolis-Num.d	Peonidin-3-o-glucoside	Sample	741	148.2484	ng/mL
SK_Propolis-Num.d	4-OH-Benzoic Acid	Sample	40668	2686.7663	ng/mL
SK_Propolis-Num.d	Epicatechin	Sample	145	0.0000	ng/mL
SK_Propolis-Num.d	Epigallocatechin Gallate	Sample	1	0.0000	ng/mL
SK_Propolis-Num.d	Caffeic Acid	Sample	2521239	49483.7460	ng/mL
SK_Propolis-Num.d	Vanillic Acid	Sample	1471	3004.1509	ng/mL
SK_Propolis-Num.d	Syringic Acid	Sample	11	0.0000	ng/mL
SK_Propolis-Num.d	Vitexin	Sample	8243	44.1778	ng/mL
SK_Propolis-Num.d	Naringin	Sample	74	0.0000	ng/mL
SK_Propolis-Num.d	Ellagic Acid	Sample	620	99.0671	ng/mL
SK_Propolis-Num.d	Hesperidin	Sample	3137	249.7517	ng/mL
SK_Propolis-Num.d	p-Coumaric Acid	Sample	1566896	31124.9127	ng/mL
SK_Propolis-Num.d	Sinapic Acid	Sample	24	0.0000	ng/mL
SK_Propolis-Num.d	Taxifolin	Sample	94743	2204.5354	ng/mL
SK_Propolis-Num.d	Ferulic Acid	Sample	179250	41525.3079	ng/mL
SK_Propolis-Num.d	Rosmarinic Acid	Sample	3708	920.3959	ng/mL
SK_Propolis-Num.d	Vanillin	Sample	2229	329.2110	ng/mL
SK_Propolis-Num.d	Myricetin	Sample	999	0.0000	ng/mL
SK_Propolis-Num.d	Resveratrol	Sample	0	0.0000	ng/mL
SK_Propolis-Num.d	Luteolin	Sample	292993	2731.7785	ng/mL
SK_Propolis-Num.d	Quercetin	Sample	479234	57654.7720	ng/mL
SK_Propolis-Num.d	Apigenin	Sample	68693	589.8101	ng/mL
SK_Propolis-Num.d	Naringenin	Sample	16604	234.4466	ng/mL
SK_Propolis-Num.d	Isorhamnetin	Sample	677420	4778.3925	ng/mL
SK_Propolis-Num.d	Chrysin	Sample	5384	88.2617	ng/mL
SK_Propolis-Num.d	Galangin	Sample	48417	5075.5507	ng/mL
SK_Propolis-Num.d	Curcumin	Sample	4	0.0000	ng/mL
QuantReport_ESTD_Complete_B_06_00.xlsx			Printed at: 5:26 PM on: 6/16/2023		
